# Data from in Rod We Trust – the Evaluation of a Virtual Rod and Frame Test as a Cybersickness Screening Instrument

**DOI:** 10.5334/jopd.161

**Published:** 2026-10-09

**Authors:** Judith Josupeit, Leonore Greim

**Affiliations:** 1 TU Dresden, DE; 2 TU Chemnitz, DE

**Keywords:** Virtual Reality, Data Logging, Unity, Cybersickness, Ecological Validity

## Abstract

We present data from a virtual Rod and Frame Test (RFT) used to screen for cybersickness and data from a gamified spatial orientation task (City) used as a reference for inducing cybersickness. Data include RFT and City performance, head tracking, self-reported cybersickness symptoms, and system logs. As cybersickness experiments are stressful, reusing this data reduces participant burden and supports ethical research. The dataset enables replication, further analyses (e.g., postural stability), and joint analyses. We further describe the open-source code for the Virtual Reality (VR) applications that make the logging rationale transparent. Moreover, this promotes efficient sampling, customizable scenarios, and methodological transparency.

## (1) Background

Virtual reality (VR) applications can trigger symptoms ranging from general discomfort to disorientation and nausea (LaViola, 2000). These symptoms are subsumed under the term cybersickness. While cybersickness resembles motion sickness (MS, Reason & Brand, 1975), these symptoms are exclusively visually induced (VIMS, Muth et al., 2018). Although not without controversy (Stanney et al., 1997), established working models for MS can be applied to explain VIMS: most models attribute the condition to a mismatch between various afferences (e.g., visual, proprioceptive, and vestibular; Kennedy, 1975). A refinement of this model stresses the importance of verticality for the genesis of MS (Bles et al., 1998). Verticality perception differs between individuals in its dependence on visual information; individuals with a higher visual dependence might have a higher awareness of conflicting cues when motion is visually displayed, yet lack a physiological counterpart (Barrett & Thornton, 1968). Conveyed to VR, this would be the case when controller locomotion is used and, therefore, seen but not physically felt.

To investigate the relationship between individual verticality perception and cybersickness, the dataset includes performance measures from a virtual Rod and Frame Test (RFT) — original physical setup by Witkin and Asch (1948). In the RFT, participants adjust a tilted rod to their perceived verticality while ignoring a distractor; that is, a surrounding tilted frame. Individuals with higher visual dependence should display a weaker performance in this test.

To correlate these performance data with cybersickness, participants also completed a gamified visuospatial orientation task in a virtual city (City) using controller locomotion. The dataset captures subjective cybersickness ratings from the Simulator Sickness Questionnaire (SSQ, Kennedy et al., 1993) and objective RFT performance measures. Crucially, the experimental design accounted for construct stability by administering the RFT either before or after the City. Additionally, the dataset provides access to in-game data in the form of head tracking data and controller input tracking. These objective streams are generated directly and periodically through the Unity application without the need for external tracking technologies (onUpdate, Unity, 2019a). The sampling logic is part of the research data and, therefore, also made transparent in this descriptor.

This data descriptor facilitates the reuse of research data to address both ethical and scientific concerns. Ethically, the induction of cybersickness represents a stressful event for participants; therefore, reusing this data reduces the need for future experiments that would expose additional participants to stressful stimuli. Scientifically, as cybersickness symptoms are VR application specific (Josupeit, 2023), additional information about the research data is beneficial for transparency and for classifying results in integrative works such as meta-analyses. By providing open data and open methods, this descriptor allows researchers to analyze the sampling process and adjust the setup for further developments without requiring additional calibration or software. In contrast to the use of external tracking technologies, which would require additional calibration and timestamp synchronization, software and hardware, the sampling logic explained in the current paper allows for easy access to insight into the sampling process. For further information regarding the original research, the corresponding research paper should be considered (Josupeit, 2024d).

## (2) Methods

### 2.1 Study design and Paradigm

The study used a repeated-measures design. Participants were randomly assigned to one of the two starting conditions (RFT or City), ensuring that each participant completed both tasks in a counterbalanced order. Participants completed both the RFT and City on the same day, with a 10-minute break in between. This design increases statistical power and enables testing of order effects. Both dependent variables (cybersickness and RFT performance) were sampled multiple times. The dataset includes all time-stamped responses, task performance logs, and processed data.

The two experimental conditions are briefly introduced; for a detailed description of the applications, refer to section *2.5.3 VR Applications*.

#### 2.1.1 Virtual RFT

The RFT was based on the paradigm of Fiori et al. (2014) but conveyed the peculiarities of digital representations like VR (Docherty & Bagust, 2010). Further perception of proprioceptive cues was limited as the task was conducted in a standing setup. Participants were presented with a tilted frame (±33° or 0°) surrounding a rod that was rotated independently (±22° or ±11°; see Figure 1). Using a controller, participants adjusted the rod to appear vertically aligned (i.e., perpendicular to gravity) under three conditions. (1) Compatible trials: frame and rod rotated in the same direction, (2) Incompatible trials: frame and rod rotated in opposite directions, (3) Neutral trials: no frame rotation.

**Figure 1:**
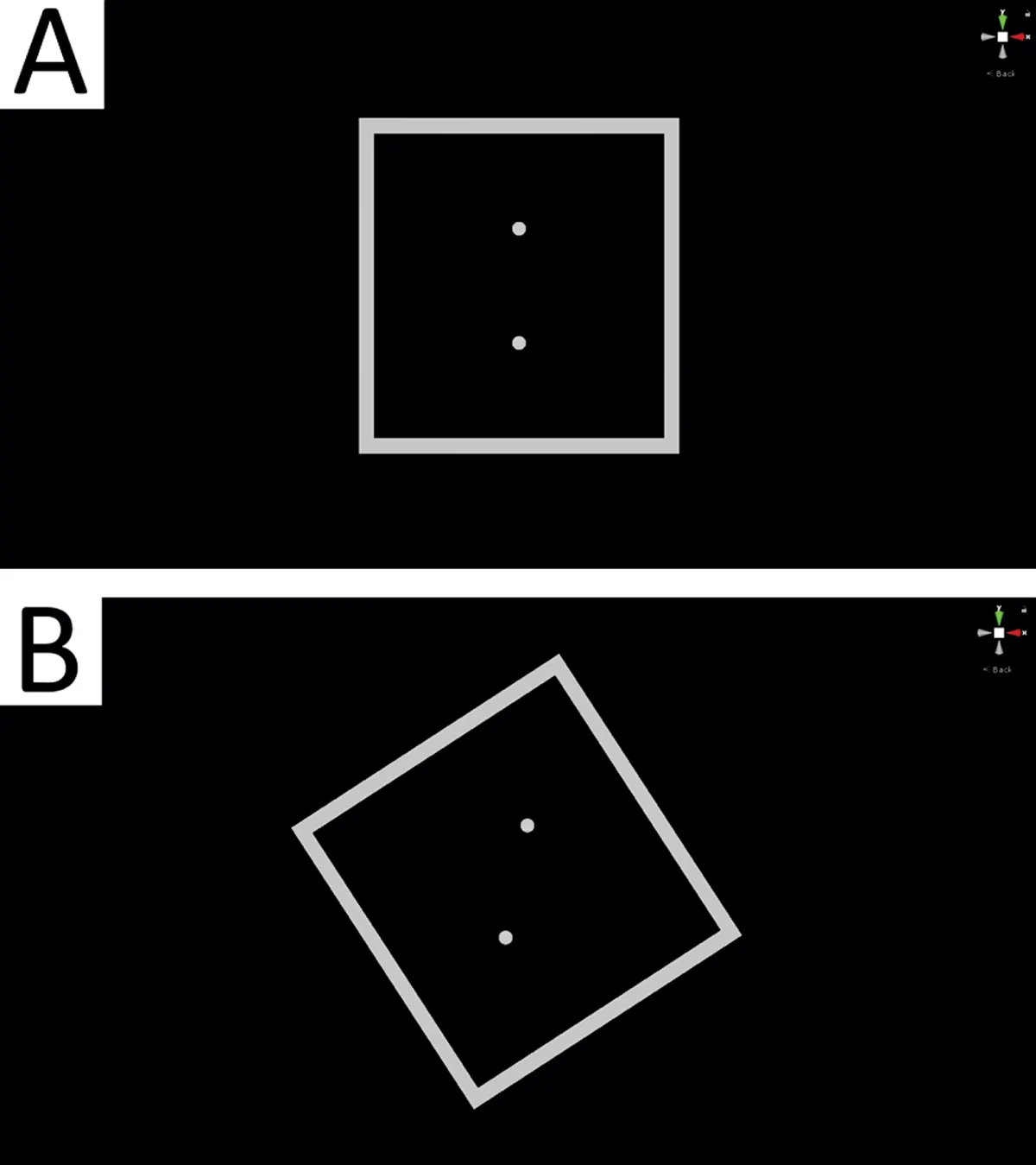
Screenshot of the virtual RFT in Unity Editor. *Note*. **A)** Tutorial trial: rotation of the rod and frame absent. **B)** Experimental trial: clockwise rotations of the rod (11°) and the frame (33°).

To ensure reliable results, three iterations of 12 varying constellations of the rod and the frame (Fiori et al., 2014) and two test trials were used, resulting in 38 total trials. The task was self-paced, with participants completing trials at their own speed. The average duration across participants was 7.00 minutes (*SD* = 2.20), ranging from 2.87 to 13.00 minutes. The primary variable recorded was the rod angle per trial.

#### 2.1.2 City

In the City condition, participants were tasked to find up to 11 virtual checkpoints by navigating in an urban environment using controller locomotion (Figure 2). The task was designed to induce motion-related cybersickness through continuous (self-paced) locomotion. Again, the perception of proprioceptive cues was limited as the task was conducted in a standing setup. The exposure time was standardized to 10 minutes. Even though a variety of variables was recorded, for the research paper the number of checkpoints reached was the only variable analysed (*M* = 7.57, *SD* = 1.84.; range 1 to 10).

**Figure 2:**
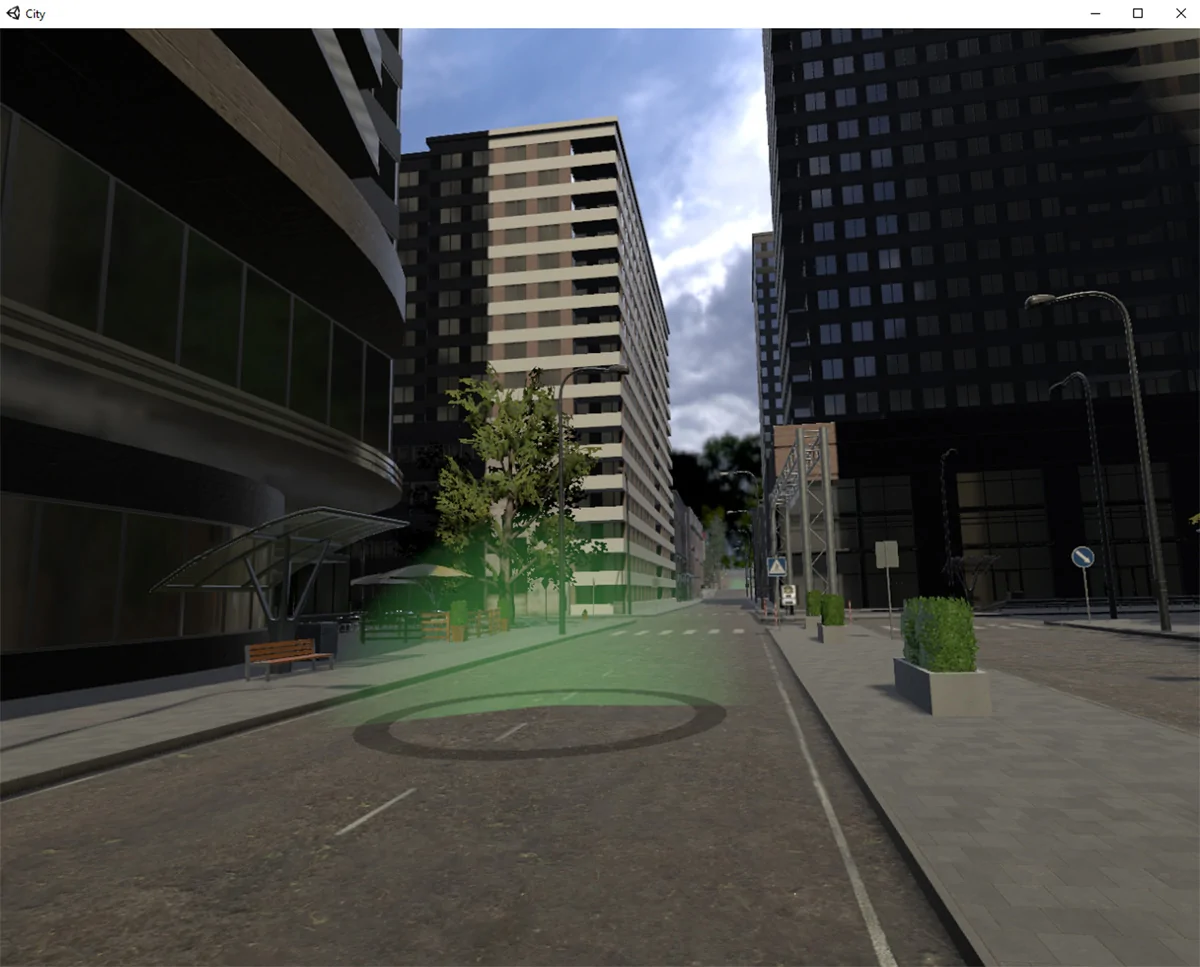
Starting point in the City in windowed mode, experimenter’s view in the build Application. *Note*. A virtual checkpoint is directly in front; another checkpoint is visible in the distance.

### 2.2 Time of data collection

Data was collected from November 2022 to February 2023.

### 2.3 Location of data collection

Data was collected at the virtual reality lab, chair of Engineering Psychology and Applied Cognitive Research, at TUD Dresden University of Technology, Germany.

### 2.4 Sampling, sample and data collection

#### 2.4.1 Sample Size Planning and Power Analysis

Based on a significance level of α = .05, a statistical power of 80%, and a conservative effect size estimate (*r* = .30), an optimal sample size of *n* = 67 participants was determined for a bivariate normal model of a correlation using G*Power, version 3.1.9.4 (Faul et al., 2007).

#### 2.4.2 Participant Recruitment and Inclusion Criteria

Inclusion criteria were: (1) normal or corrected-to-normal vision (including stereo acuity), this prerequisite was tested with two standard tests for visual and stereo acuity; (2) being of legal age (≥ 18 years), (3) being fluent in German, and (4) no history of epilepsy, migraine, or pregnancy.

In total, 87 participants volunteered for this study. Of these, six participants had to be excluded from the final analysis due to visual impairments. However, their data was collected and can be found in the data repository, because these students were receiving course credit and thus were allowed to participate despite failing the eligibility tests, due to institutional policies. Data are flagged for exclusion in analyses. In addition, data sets are incomplete for four others because they experienced severe symptoms of cybersickness (more details in section 2.4.5 Dependent Variables), and one more because of technical issues. All exclusions are documented in the experimenter’s protocol, including the reasons for exclusion.

#### 2.4.3 Analytical Sample

The analytical sample of this descriptor has 76 participants, with 28 males and 48 females. The age of the participants ranged from 18 to 55 years (*M* = 26.17 years, *SD* = 7.38). 23 were requiring a visual aid. 45 had no prior VR experience; 15 had less than 30 min; 15 had between 30 min and 5 hrs, and one had more than five hrs of VR experience.

#### 2.4.4 Demographic Questionnaire

A demographic questionnaire contained questions regarding age, gender, and whether the participant had been more prone to dizziness or nausea than usual over the last 6 days, including the current day. Additionally, information was collected regarding the requirement of a visual aid and whether they had prior experience with VR, including the extent of that experience if applicable.

#### 2.4.5 Dependent Variables

##### 2.4.5.1 RFT Performance

Performance in the RFT was quantified using four complementary indices:

1. the Mean Absolute Deviation, which is the average absolute angular deviation from true vertical (0°) across the compatible and incompatible trials (Maneuvrier et al., 2021; Reger et al., 2003; Witkin & Goodenough, 1977). This index captures overall perceptual error but does not differentiate between the real frame dependency and a participant’s internal response consistency.
2. the Constant Error, reflects a participant’s consistent bias (e.g., tendency to over-rotate left or right), independent of frame orientation or the rod starting position (Nyborg, 1974).
3. the Frame Effect: calculated by subtracting the constant error from the mean right-tilted frame condition, reflecting the degree to which the frame influences perceived verticality (Nyborg, 1974).
4. the Frame Influence: Computed as the difference between the mean angle error in neutral trials and the mean error in compatible/incompatible trials, quantifying the magnitude of frame-induced distortion (Fiori et al., 2014). The raw data for the derived performance measures were logged on Update() (Unity, 2019a) around 60 Hz (see for source code: Josupeit, 2024e; section 2.5.3 VR Applications).

##### 2.4.5.2 Cybersickness

For assessing cybersickness, two questionnaires were utilised. First, a long symptom questionnaire, the SSQ, with 16 items designed to quantify symptoms of VIMS, was deployed (Bimberg et al., 2020; Kennedy et al., 1993). The participant rated the extent to which each symptom applied to them on a scale from 0 (none) to 3 (severe). The questions were read aloud by the experimenter to ensure consistency, while avoiding giving external reference points for spatial calibration in the form of vertical and horizontal text information. The SSQ was administered before VR exposure (baseline) and after VR exposure (post-VR) in both conditions.

Secondly, during the VR exposure, the 11-point MISC (Misery Scale; Bos et al., 2005) was administered, allowing for a fast assessment of the participant’s current well-being, on a scale from 0 to 10. Possible responses are divided into a four-tiered subscale: from no problems (0), mild discomfort (1), to specific symptoms (2–5), and escalating discomfort (i.e., nausea 6–9), up to vomiting (10). In line with the literature, a termination condition indicating nausea (MISC ≥ 6) was set to ensure the participant’s safety (Kuiper et al., 2020). Both SSQ and MISC were translated into German by the three experimenters individually. Then a final translated version was generated and re-translated into English to ensure reliable translations, as no official translation was available. Assessment intervals were every 2 min during the exposure to the City and after one set of 12 trials in the RFT.

Δ-Cybersickness scores were calculated as the difference between post-VR SSQ/MISC scores and baseline scores for each participant and condition. After meeting the preconditional hypothesis of higher scores post-VR compared to baseline, following the suggestion of previous research (Josupeit, 2023). This metric reflects the change in symptom severity due to VR exposure and is included in the dataset as a derived variable.

#### 2.4.6 Procedure

To follow the procedures, the reader is referred to Figure 3.

**Figure 3:**
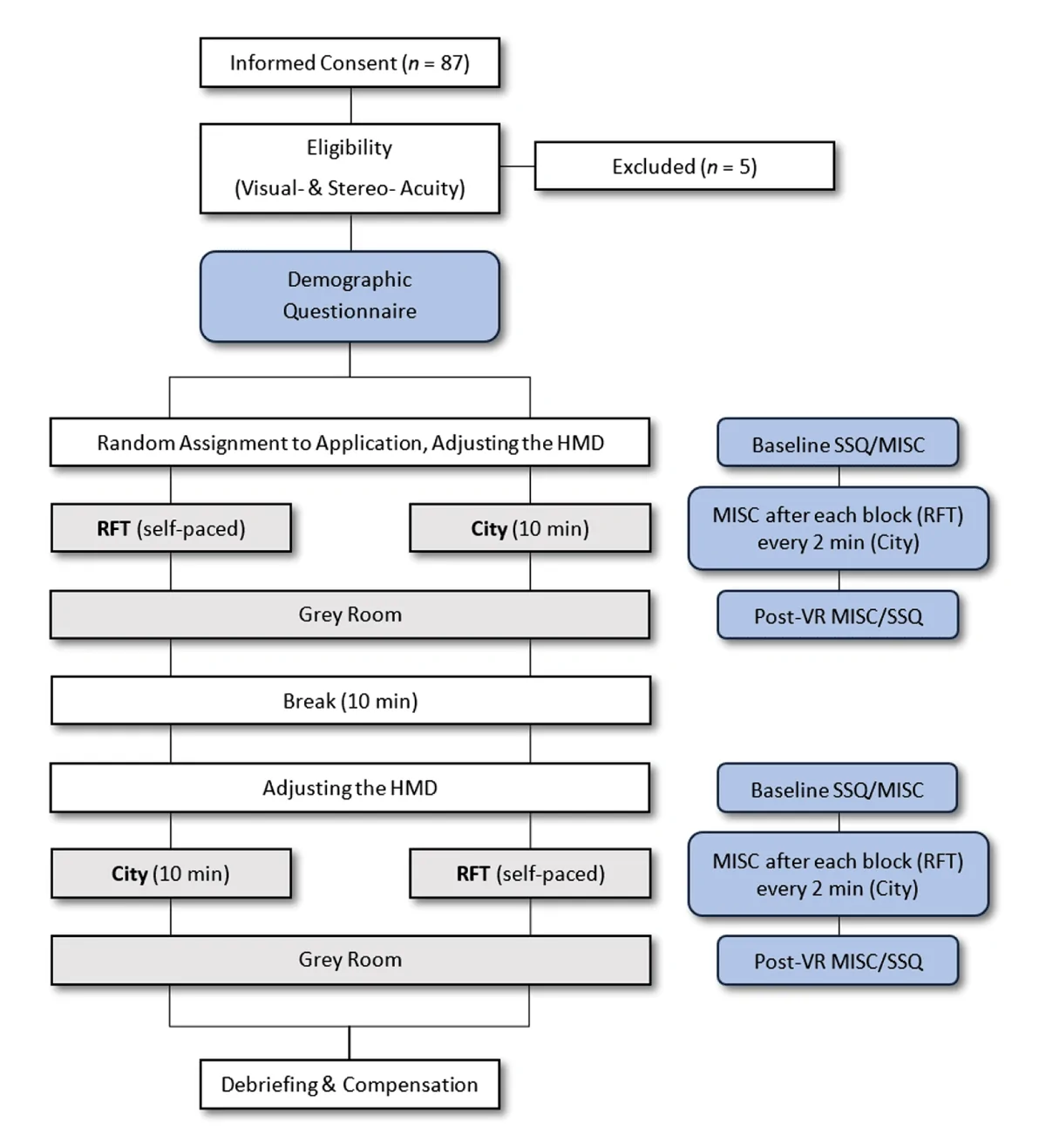
Flowchart of the experiment. *Note*. Questionnaire assessments are highlighted in blue, the VR environments in light grey, and other procedures in white.

First, the participant was provided with an information sheet along with a consent form. Once they had signed the form, the tests for visual acuity were conducted to ensure that participants had normal (or corrected to normal) vision (see section *2.5.2 Prerequisite Tests for Visual Acuity)*.

After passing both visual tests, participants completed a demographic questionnaire. Thereafter, the participant was presented with a print-out version of the two questionnaires that would be asked verbally during the experiment (i.e., SSQ and MISC). They were instructed to read them and to clarify any misconceptions. Subsequently, the controller and the handling of the relevant keys of the controller, which are the trigger and trackpad, were explained.

Then, the participant was instructed to stand upright in a designated position in the laboratory. The VR headset was fitted based on the participant’s individual head shape. Participants adjusted the interpupillary distance and the eye relief themselves during the SteamVR loading screen based on subjective visual clarity. Considering physiological aspects for VR research has been discussed as a crucial aspect in previous works (Maneuvrier et al., 2023; Stanney et al., 2020).

The controller was handed to the participant’s dominant hand, and the Unity application was launched, displaying either the RFT or City as the first VR application in random order. Then, the baseline assessment of the SSQ and the MISC was conducted in the grey room, and the participant was asked if they felt capable of continuing.

Subsequently, the task instructions for the respective experimental task were given by the experimenter. While the participant engaged with VR, their actions were observed, and guidance was provided whenever necessary. This was particularly important for the RFT, as some participants confused vertical with horizontal. In addition, the experimenter paid close attention to any signs of severe discomfort and ensured that the session was terminated immediately in case symptoms of nausea occurred.

The MISC was administered after the completion of each block in the RFT (i.e., 12 trials each plus the two test trials) and 2 minutes in the City application. If the termination condition was exceeded, the VR exposure was quit (Kuiper et al., 2020).

At the end of each application, the participant was presented with the grey room again for the post-VR assessment of the SSQ. After the first application, there was a 10-minute break without the VR headset. The participant was offered a beverage and a snack. After the break, the second application followed with a homologous assessment rationale.

After completion of the second VR exposure, the participant was debriefed and compensated with either €10 or course credit before being dismissed.

### 2.5 Materials/Survey instruments

#### 2.5.1 Hardware/Software

A custom-built PC equipped with an NVIDIA GeForce RTX 2070 GPU and an Intel Core i7-9700K processor rendered the VR application. The operating system was Windows 10 (64-bit) in German language settings. As the VR headset, the HTC Vive (HTC Corporation, Taiwan, 2018) was used.

For the participant, interaction with the VR environments was enabled through the controller of the HTC Vive. The key bindings for the input were equivalent for the left and the right controller (mirrored mode). Because only one controller was required for interaction with the VR applications, one controller was permanently charged as a backup to allow for a smooth experimental procedure on days with extensive use of the controller. Moreover, the experimenter started data logging of the application and was able to reset and quit the application upon request by using the keyboard and mouse of the rendering PC.

The calibration procedures used the Steam VR room setup to determine ground level, the rendering PC position, and the play area, a virtual room measuring 3.42 x 5.42 m. The dashboard application OVR Advanced Settings was used to disable the Steam VR safety chaperone for the VR applications (OVRAS Team, 2016). Usually, the chaperone displays the boundaries whenever a user exits the calibrated space, which would have facilitated orientation in the VR application.

The first author used the Unity game engine (v2019.1.11f1) to develop both VR applications, which are explained in more detail in the upcoming section *2.5.3 VR Applications*. The custom key bindings for interaction with the virtual objects and motion translation were enabled through the Steam VR Asset Version 2.3.2 (Valve Corporation, 2023).

All game objects and materials for the RFT were custom-made using the Unity-inherent functionalities. The game objects in the City were extracted from the Windridge City Asset and enhanced with custom-designed features such as virtual checkpoints and a custom-made cylinder using Blender.

A touchscreen tablet was used to complete the demographic questionnaire in LimeSurvey (Version 5.6.44). During VR, the experimenter also used this questionnaire for giving standardised instructions and logging the answers to the cybersickness questionnaires.

#### 2.5.2 Prerequisite Tests for Visual Acuity

As an eligibility check for a participant, visual acuity was tested. The Freiburg Visual Acuity Test (FrACT, v 3.10.5) was applied with the exclusion criterion of logMar < 0 (Bach, 2007, 2021). The Landolt-Cs of the FrACT were displayed on a second laptop, a Lenovo Thinkpad (E570) with a 15.6” screen diagonal and an observer distance of 2.43 m, which allowed for a detection of a maximal decimal visual acuity of 2.38. A USB numeric keypad with the Landolt-C orientations affixed to the respective numbers was used for the participant’s entries. The second visual acuity test was the random dot stereo acuity test (Vision Assessment Corporation, IL USA, 2007). Participants with insufficient stereo acuity (i.e., ≥ 50 seconds of arc) were excluded to ensure the participant was able to perceive the VR as intended and to reduce the likelihood of eyestrain (Deepa et al., 2019). For both tests, the participant was instructed to guess whenever they were uncertain and was allowed to use visual aids if necessary for the tests and during the VR exposures.

#### 2.5.3 VR applications

For a deeper insight into the applications, the GitHub repositories containing the built versions (Josupeit, 2024a, 2024b) and the source code (Josupeit, 2024c, 2024e) should be considered. All of them have an overview in their Readmes.

To reduce the likelihood of the experimenter’s error, applications were built in windowed mode instead of using the development versions in Unity Editor. In both applications, an input field and a grey waiting room were used for data labelling and asking the cybersickness questionnaires. In both applications, an individual log file in CSV format was automatically created in the application’s data folder after the experimenter had entered the participant’s code in the input field and confirmed the entry.

##### 2.5.3.1 Data Logging

Since much information regarding the log files is identical for both VR applications, the following paragraph introduces the general data logging logic. VR application-specific information is explained in the designated preceding sections. The associated script is called *Output.cs* and generates a CSV file for each VR exposure individually. The designation of the logged file follows the format: date in long format (Weekday, DD. Month YYYY) followed by the system time in seconds (“HH-mm-ss”) when the application was instantiated. It was necessary to use “–” as the separator between hours, minutes, and seconds, because “:” is not valid for file names using Windows. Designation with the system time was absent for the first seven participants. This information was added as redundancy to prevent overwriting in case the experimenter mistakenly confirmed an entry with a participant’s code that had already been used that day. This entry is followed by the participant’s code (consecutive numbers from 1 to 87; for example, Mittwoch, 21. Dezember 2022_15-29-14_51). Rearranging the labelling or adding further information (e.g., name of the application) would, of course, be possible in future work.

The experimenter entered the participant’s code into the input field and confirmed the entry with Enter on the numpad. This set the input field to non-interactable and started the data logging. Sampling of the data was following the pre-set frame rate (onUpdate) of the application (Unity, 2019a). For the build applications, it is almost 60Hz. Data logging ended automatically once the experimenter quit the application by clicking the right mouse button.

An unusual delimiter “/” is used to separate the values in the CSV file. This approach was motivated by the difference in decimal separators between the operating system (using German conventions) and Unity (using English conventions). To facilitate the use of the log files in this unusual format, the reader should consider the first lines of code in the pre-processing script in the repository (see *2.5.4 Data Preprocessing*).

For future projects, it might be advisable to use a uniform convention to avoid confusion based on different language conventions. Nevertheless, the solution, equally to changes from “:” to “–” for the file name, specifies how incompatibilities have been dealt with in the current approach; for reasons of transparency, the raw data are left unaltered. The data sampled includes the system time in 10^–5^ s, as well as head-related data (camera position and rotation in x,y,z-coordinates) and behavioural data (controller input in x,y-coordiantes).

##### 2.5.3.2 Details: RFT

The virtual RFT started with a tutorial trial (Figure 1A) in which the participant was familiarised with the handling of the controller (rotating objects with the trackpad and confirming entries with the trigger). The rod was realised as two dots that were attached to the edges of a translucent rectangle (Docherty & Bagust, 2010). This rectangle was the actual rod for which the rotational value of the z-axis was logged in the CSV file every frame (“Z_Rod” in the log file). The participant was instructed to envision a line connecting the two dots to a rod. Once a press of the trackpad was recognised (on state(), Valve Corporation, 2023), the turning of the rod began: The direction was determined by the position of the participant’s thumb on the trackpad. Touching the left side of the trackpad resulted in negative values leading to clockwise rotation, and vice versa for the right side, with increments of 0.1° whenever the turning function was executed. Figure 4 illustrates the coordinate system that corresponds to the values in XY_Controller_Input on a stylised trackpad.

**Figure 4:**
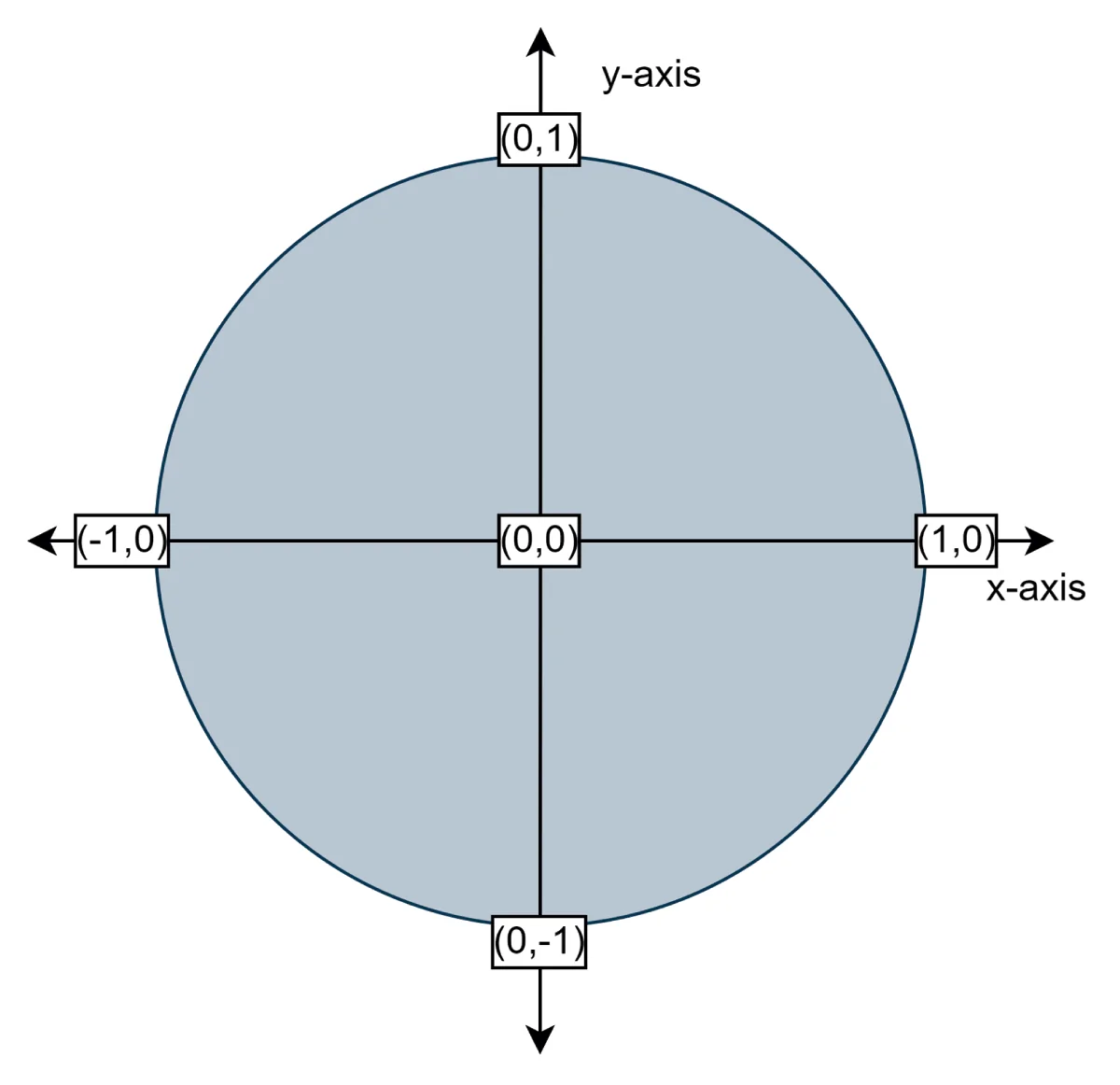
Schematic illustration of coordinates associated with input on the trackpad. *Note*. Controller Input has an x and y coordinate; the x coordinate was relevant for the RFT application, while the y coordinate had been relevant in a previous version of the City application.

To start the first trial, the participant needed to press the trigger. The participant’s task was to estimate verticality realised by the rod in the context of irrelevant surrounding information in the form of the misleading orientation of the frame (Figure 1B). The z-axis rotation of the frame is called “Z_Frame” in the log file. The participant was encouraged to minimise their head movement and to keep their head straight during the task. In addition, head movement data were sampled to control for participants with extensive movement. However, this information has resolution limits compared to concurrent motion capture systems.

To achieve the constellations, both the rod and the frame had pseudorandomized starting orientations along the rotational z-axis.^1^ In addition to the 38 trials, three blank trials were added after each complete set of constellations (additional trials 15, 28, and 41). During these trials, the experimenter asked the MISC questionnaire (see section *2.4.5 Dependent Variables*). Because the subjects were completely dark-adapted in the RFT, a blank screen (black) was also inserted between the trials to reduce the influence of afterimages from previous trials. In addition to the blank screen, a customised material (Josupeit, 2024e) was used to reduce the contrast between the illuminated rod and frame and the black background.

For confirmation of the current estimate of the subjective verticality and initialisation of the next trial, the participant needed to press the trigger, but trials could be reset upon request by having the experimenter press escape on the keyboard.

The data log file contains the following additional information: “Trialnumber” is the integer that refers to the current constellation. Unlike the counter “Trigger”, the “Trialnumber” is affected by the corrective trials whenever the Escape button is pressed by the experimenter. “Trigger”, in contrast, counts the number of trigger presses by the participant. For technical reasons, the counter “Trigger” is activated at the beginning of the registered button press when the trigger is pressed (GetStateDown()), while the counter “Trialnumber” is activated at the end when the trigger is released again (GetStateUp(), Valve Corporation, 2022). While “Z_Frame” is unaffected by “X_Controller_Input”, “Z_Rod” is rotated based on the value in the previous column. Both “Z_Rod” and “Z_Frame” change upon “Trigger” counts until “Trialnumber” 41 is reached.

##### 2.5.3.3 Details City

The starting point (Figures 2 and 5) was determined to be a central point on the terrain (Unity coordinates 115, 542.7, –271). Because the terrain was quite hilly, the y-axis is relatively high (for an illustration, an interactive 3D scatterplot is accessible in the data repository, Sievert, 2020).

**Figure 5:**
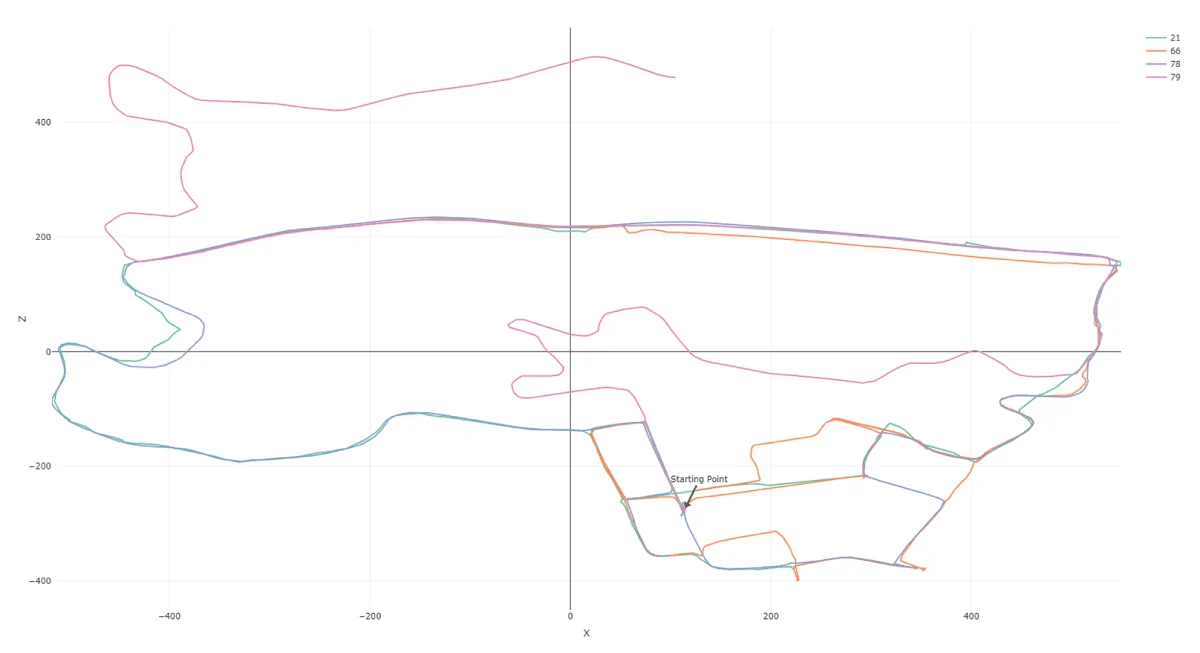
Reconstructed paths of four participants in a bird’s-eye view. *Note*. The black arrow defines the starting point, and the four exemplary line graphs illustrate the overlap and differences in search strategies of these four participants.

To induce cybersickness deliberately, artificial locomotion, different terrain heights, distinct camera settings (i.e., orthographic), and a skybox that rotated in the opposite direction to the participant’s head rotation were incorporated. This way, there was no fixed point on the horizon on which a participant could focus.

As expectations influence the occurrence of symptoms and thus the results of the study, the participant was provided with a cover story claiming the main goal was to test visuospatial orientation in the virtual environment (Young et al., 2007).

To keep the participant engaged in exploring the City, gamification was deployed: The participant was tasked with collecting virtual checkpoints, represented by green-glowing circles distributed throughout the City. Once a checkpoint was reached, the material of the checkpoint changed from green to yellow (onTriggerEnter(); Unity, 2019b). Once it was passed (onTriggerExit(); Unity, 2019b), it was destroyed (Checkpoint.cs). Collision detection was run for each checkpoint individually. Checkpoints were all duplicates of another, containing the following properties: the tag “Checkpoint”, a collider, and the script. In the log file, game objects with the tag “Checkpoint” are counted. Whenever the number of checkpoints decreases because of successful collection the variable “Checkpoints_Reached” increases.

While there are other ways to realise linear acceleration in VR (e.g., using Steam VR’s player prefab), the code for the locomotion in the City was custom-made (VR_Controller.cs). The locomotion was inspired by a series of YouTube tutorials, no longer publicly available (Connell, 2019a, 2019b, 2019c, 2019d), but also contains further customization for using the controller locomotion in the City. This had the advantage of direct manipulability of trackpad sensitivity, locomotion speed, and direction, as well as logging the updated camera data (i.e., virtual camera position and physical head rotation) during locomotion with no need to deactivate further bindings. Locomotion was realised whenever the participant touched the trackpad. The movement occurred at a maximum speed of 2.1336 m/s. To facilitate handling, only forward locomotion was implemented in the direction of the current head rotation. For changes in the direction of the movement, the participant needed to rotate around their yaw axis.

As for the virtual RFT, the escape button served the purpose of resetting. However, in the City, the reset meant a reset of the camera to the starting position, without resetting the checkpoints that had been collected. For checking whether reset was used during VR exposure reconstruction of the path taken by the participant based on the virtual camera position (“X, Y, Z_Head_Position”) is useful. Figure 5 illustrates the paths four of the participants took during the 10 min of VR exposure; in addition, a supplementary interactive figure is provided in the research data repository.

In addition, the logfile contains the following information: “Button_Void” is the integer that refers to the activation and deactivation of the grey room. As the script for the grey room uses a Boolean condition, even numbers mean the grey room is activated, while odd numbers mean that the city environment is displayed. In addition, the “Controller_Input” contains information regarding the presence or absence of the virtual movement.

#### 2.5.4 Data Preprocessing

The pre-processing of the Unity log files included: Firstly, the deletion of test datasets and other irrelevant records (e.g., very small datasets). Secondly, individual cleaning and processing of each participant’s dataset for the RFT and City using an R script.

New variables were created: For the RFT data, the following values were extracted:

# Constel – comprised code of the starting constellation of rotations for rod (–22, –11, 11, 22) and frame (–33, 0, 33), format “rod_frame”, e.g. “–22_–33”

# Trialnumber – counter for activated trials, higher when a specific trial was revised

# Z_Rod – rotation angle of the rod in global Unity-coordinates z-direction at the end of the trial

# Z_Frame – rotation angle of the frame in global Unity-coordinates z-direction

# Time – time stamp (format HH:mm:ss) of first registered controller input for each trial

# Time_Abs – absolute trial duration in seconds

# VP_No – participants code

# Block – blocking trials 1–15, 16–28, and 29–40 in blocks 1, 2, and 3

# Condi – condition of the rod and frame constellations depending on alignment: compatible, neutral, or incompatible

For the City data, the participant’s code and the maximum number of checkpoints reached for each participant were pre-processed.

The LimeSurvey data encompass the demographic information, as well as their responses to the cybersickness questionnaires. The unfiltered data exported from LimeSurvey underwent modifications for the filtered version, ensuring that only one row exists for each participant.

In the unfiltered data, there may be two rows for a single individual. During the experiment, Wi-Fi connectivity issues occurring during the breaks interrupted the survey prematurely and required a restart of the LimeSurvey poll. Additionally, some corrections for demographic data were made based on the experimenter’s protocol and incorporated into the filtered version.

Within the LimeSurvey data, for the SSQ and MISC variables in the City, there were two versions: one where the City application comes first, and another where the City application follows the RFT application. In the merged version, these are combined into single variables, resulting in only one instance of each variable for the City questionnaire data. Furthermore, responses in LimeSurvey, initially expressed in strings (words) in the raw data, were coded with numeric values (e.g., 0 and 1 instead of “male” and “female”).

### 2.6 Quality Control

To ensure the quality of the collected data, it was necessary to confirm that participants could perceive the VR environments as intended. Therefore, participants needed to pass the eligibility tests. Another quality control check provided by the participant is the question regarding dizziness and nausea in the demographic questionnaire, allowing for verification of whether symptoms were caused by the VR exposure or pre-existing conditions.

Quality control can further be addressed from the experimenter’s point. The experimenter’s protocol was also used to document irregularities during the experiments, enabling post-hoc corrections to the data or providing reasons for exclusions of certain data sets. In addition, the experimenter had to stick to a standardized protocol that was included in the LimeSurvey questionnaire and can be accessed through the respective resource or permalink in the research data repository.

In addition, the Unity data can be checked for plausibility (e.g., see Figure 5). Understanding the logic behind the Unity data log files enables greater transparency of research data.

### 2.7 Data anonymisation and ethical issues

The study procedures adhered to the ethical guidelines outlined in the Declaration of Helsinki and received prior ethical approval from the local ethics committee at the Technical University Dresden (EK530122019). The written informed consent contained information in line with the General Data Protection Regulation (GDPR) about storing and publishing the data.

### 2.8 Existing use of data

The research paper associated with the data has been published in Josupeit, J. (2024). In rod we trust–The evaluation of a virtual rod and frame test as a cybersickness screening instrument. *PLoS ONE* **19**, Article e0313313, doi:10.1371/journal.pone.0313313.

## (3) Dataset description and access

### 3.1 Repository location

The dataset is available at OPARA [DOI 10.25532/OPARA-920] (Josupeit et al., 2025).

For the Unity source code, the reader is further referred to the following GitHub repositories:

The RFT project (opens in Unity Editor): https://github.com/JudiJ/RFT_Project

The RFT application (Unity.exe): https://github.com/JudiJ/App_RFT

The City project (opens in Unity Editor): https://github.com/FAndrees/City_Project

The City application (Unity.exe): https://github.com/JudiJ/App_City

### 3.2 Object/file name

The data is compressed into a zip folder named *VR_RFT_2022*. With the following structure

Top level:

Experimental_Protocol_Index.xlsx, containing the reasons for inclusion or exclusion of data for the analysis of the research paper (Josupeit, 2024d). Included is an index: 0 for inclusion, 1 for a borderline case, and 9 for exclusion.

Subfolders:

1. VR_RFT_RFT/: RFT Unity data (raw and pre-processed).+ pre-processing R script^2^
2. VR_RFT_City/: City Unity data (raw and pre-processed).+ pre-processing R script
3. VR_RFT_Limesurvey/: LimeSurvey data (raw and pre-processed) SSQ/MISC and demographic data.) + pre-processing R script. To overcome language barriers, all information regarding the LimeSurvey material is additionally presented in English. Moreover, the *.lss* LimeSurvey resource is available in German and English translation. In the Readme file, there is also a permanent link to the questionnaire.

For an overview, each folder contains an overarching Readme file. The R scripts for pre-processing are available and use packages for increased accessibility *here* (Müller, 2020) and for version control *groundhog* (Simonsohn & Gruson, 2023).

The _*final* files in each folder are the result of filtering and pre-processing.

### 3.3 Data type

The repository contains primary raw data, filtered data, and scripts for making the preprocessing transparent. In addition, the source code and the applications are shared in four additional repositories. The shared source is new material, not published elsewhere, and provides a deeper understanding of data logging, as well as perpetuating the paradigm with the chance for further development of custom applications, applying an equal logic.

### 3.4 Format names and versions

All collected research data are in CSV file format. For special formats, separators of the raw data from the VR environment refer to section *2.5.3.1 Data Logging* and the Readmes in the repositories.

The LimeSurvey questionnaire is in LSS format and can be opened in LimeSurvey.

The R Scripts can be opened in R.

For use of the executable files for the City application and the project, Git LFS is required. Files stored in LFS are of type .fbx, .unity, or .asset.

### 3.5 Language

The data and information on the data are stored in English. Original material is also available in an unaltered (i.e., German) format. As differences of the delimiter and decimal separators exist, it is suggested to consult the respective Readme or start with the English version of the data. In addition, as mentioned before, the file names of the data from the VR exposure are in German (e.g., Mittwoch, 21. Dezember 2022_15-29-14_51).

### 3.6 License

All data are publicly available under Creative Commons license CC-BY.

### 3.7 Limits to sharing

The data is not under embargo and does not contain any identifying information.

### 3.8 Publication date

A first version was published in 2024. However, to increase accessibility to the data for non-German-speaking users, an updated version with English translations of all material was published on 14/08/2025 to which this publication refers.

### 3.9 FAIR data/Codebook

The data is findable under this DOI https://doi.org/10.25532/OPARA-920 and in the GitHub repositories (Josupeit, 2024a, 2024b, 2024c, 2024e). It uses non-proprietary formats for as many files as possible and is described extensively via Readmes. For the research data, the Readme files and the comments within the code files provide detailed explanations of how to use and process the data. Moreover, for the questionnaire data, the codebook is provided. For a comprehensive understanding from the beginning, it is advisable to use the unfiltered data and re-run the R scripts for pre-processing. Users can filter these datasets according to their preferences using the experimenter’s protocol. Depending on the use case, it might be necessary to reconsider the indices set for the data exclusion.

## (4) Reuse potential

This paper serves as an example of how to make use of the data that VR provides without using any additional equipment. Not all of the data has been used for analysis for the corresponding research paper. Moreover, the questionnaire data could be used in factor analyses, which require an extensive amount of data, hardly collectable in one laboratory study (Josupeit, 2024d). Interesting analyses could also be derived from the head movement data (i.e., transform and position of the virtual camera). As an example, previous research used the head movement data to infer postural stability (Josupeit & Andrees, 2024). When combined with the controller input, the path taken in the virtual environment (see Figure 5) becomes reproducible at any given time. This way, patterns that overlap for certain groups of people, and the use of different strategies in the search task, become comparable. Additionally, the registered controller input could serve as a metric of confident use of the technology and be operationalised as frequencies and durations without any controller input (e.g., clustering based on self-reported previous experience).

Access to the source code allows for further development of the paradigm. In addition, the logic of the data sampling is made transparent.

This logic could also be transferred to software development for other VR applications, which are in search of efficient ways to use VR as a research tool. The current method combines the controllability of laboratory research with the freedom of applied research in the field. For example, external control by the experimenter is rather high, controlling the onset of scenes, but the participants’ task is rather unconstrained by free exploration in a virtual space that is not limited to the physical world.

## Footnotes

The order of the constellations can be inspected by combining the information of the functions TurnRod() in the Rod.cs script and TurnFrame() in the Frame.cs script.

The text file _special_cases contains the necessary changes in the pre-processing script that are attributable to corrections that required a reset of a previous trial (i.e., the experimenter pressed the escape button) and, therefore, differences in trial numbers and rod and frame constellations.
