# Supplementary material for: Data from in Rod We Trust – the Evaluation of a Virtual Rod and Frame Test as a Cybersickness Screening Instrument
Source: J Open Psychol Data. 2026 Oct 9;14:4. doi: 10.5334/jopd.161 (PMC13651891; doi:10.5334/jopd.161)
Supplement: Supplementary file 1 [file jopd-14-161-pr1.pdf]

Initial Peer Review Round for "Data from in rod we trust – The evaluation of a virtual rod and frame test as a cybersickness screening instrument"

Dear Judith Josupeit, Leonore Greim,

After review, we have reached a decision regarding your submission to Journal of Open Psychology Data, "Data from in rod we trust – The evaluation of a virtual rod and frame test as a cybersickness screening instrument". Our decision is to request revisions of the manuscript prior to reconsideration for publication.

The full review information is included at the bottom of this email. Please note that there may also be a copy of the manuscript file with reviewer comments available once you have accessed the submission account. In summary, you present a really interesting and useful dataset and we agree there is plenty of reuse potential to justify publication, however a significant range of changes would be necessary to ensure we extract as much value as possible from this. As such, I encourage you to look through the feedback below and to implement it as thoroughly and comprehensively as possible. Data papers are a little different but the detail is often the core critical issue so I hope in following their steers the paper can be more precise and practical and so we can drive more impact and use through these types of revisions. As such we are expecting substantive changes across the manuscript. I do hope this is all clear but if there's anything I can do to help please don't hesitate to reach out.

Instructions for how to resubmit your article online are pasted below. Please ensure that your revised files adhere to our author guidelines, and that the files are fully proofed prior to upload. Please also include a revised version of your article with 'tracked changes', adding comments where appropriate, to indicate the revisions made, in addition to a brief document outlining how you have responded to the reviewers' requests.

If you have trouble processing the revisions, our Help Center (https://help.u-community.io) or downloadable PDF (https://bit.ly/Author-Guide-OJS-3) may be able to help. If not, please get in touch and we'll be happy to help.

Please also ensure that all copyright permissions have been attained for any figures/tables you have included.

Please could you have the revisions submitted with two months. If you cannot make this deadline, please let us know as early as possible.

Kind regards,

Prof Thomas Rhys Evans

Reviewer 1:
Recommendation: See Comments

Comments to the author(s)

Abstract
+ the abstract reads a bit disjointed – try talking about what's in the data and end with why it's useful.

Introduction
+ While the first paragraph introduces all relevant concepts, the progression between them is not fully explicit. The theoretical discussion of cybersickness and motion sickness transitions rather abruptly into verticality perception and the RFT, leaving the conceptual link between these elements underdeveloped.
+ The focus on qualifying the RFT as a screening instrument is misaligned with the stated purpose of the manuscript as a data descriptor. These methodological validation arguments are more appropriate for an accompanying methods paper than for a dataset publication.
+ The other two paragraphs are ok but also feel a bit disjointed – try focusing on 1) what is the phenomena of interest, 2) how was it measured, 3) why reuse of the data is useful which may help with flow.

Methods:
+ The paragraph refers to experimental conditions (City, RFT), an order manipulation, and a repeated-measures design without prior explanation. For a data descriptor, these elements need to be explicitly defined in terms of what was administered, what was measured, and how this is reflected in the dataset.
+ The description relies heavily on procedural shorthand (e.g., "City," "RFT," repeated-measures design, Δ-cybersickness scores) without first defining the tasks, their sequence, or their function in the study. For a data descriptor, these elements should be explicitly described in concrete terms rather than assumed as background knowledge. The description relies heavily on procedural shorthand (e.g., "City," "RFT," repeated-measures design, Δ-cybersickness scores) without first defining the tasks, their sequence, or their function in the study. For a data descriptor, these elements should be explicitly described in concrete terms rather than assumed as background knowledge.

Sampling
+ This paragraph introduces complex inclusion and exclusion contingencies but does not clearly specify which participants are actually represented in the released

dataset. For a data descriptor, the final analytic sample and the criteria used to determine inclusion in the dataset should be explicitly stated.
+ What does passing visual tests mean?
+ The Materials section contains extensive implementation-level detail (Unity versions, camera modes, lighthouse distances, shader choices, asset sources, etc.) while omitting a concise data-level description of what variables are produced by each instrument. This makes it difficult to understand what the dataset actually contains. The focus should shift from how the VR environments were engineered to what data streams and variables were recorded and how they appear in the released files.
+ Neither the City task nor the RFT is described in terms of reusable task primitives (stimulus, response, timing, and output variables). Instead, they are described procedurally and narratively. For a data descriptor, each task should be introduced with a clear summary of:
• What participants did
• What responses were recorded
• What variables were produced
• Which files contain those variables

+ The SSQ and MISC are discussed primarily in terms of psychometric justification and termination criteria rather than as concrete data objects. For reuse, the manuscript should clearly specify which questionnaire items, scales, time points, and derived variables are present in the dataset and how they are encoded.
+ Sections 2.5.4–2.5.4.3 emphasize logging architecture, Unity execution logic, and implementation details but do not provide a clear data schema. A table enumerating variables (name, type, units, meaning, source task) is necessary for practical reuse.
+ Dataset inclusion decisions are deferred to the experimenter's protocol. This prevents readers from determining the final analytic sample from the manuscript itself. A data descriptor must explicitly define which participants and observations are included in the released dataset.
+ In its current form, the manuscript provides exhaustive procedural detail but insufficient dataset-level abstraction. The paper would benefit substantially from being reorganized around the structure, contents, variables, and reuse of the dataset rather than around laboratory execution and Unity implementation details.

Reviewer 2:
Recommendation: Revisions Required

Comments to the author(s)

Dear authors,
It was very interesting to read your manuscript describing the dataset from "In rod we trust–The evaluation of a virtual rod and frame test as a cybersickness screening instrument". While the method is well described and the data is accessible, I have a few recommendations:

A1. I believe that there is a minor discrepancy in your sample size reporting. In this manuscript, you have mentioned how data was initially collected from 87 participants and excluded 5 of them. However, in the published research paper associated with this manuscript, there were 6 initial exclusions. I would recommend updating the manuscript as well as Figure 1.

A2. Under sampling, you mention how “some additional exclusions were necessary due to technical difficulties and participants reaching the termination criterion”. The published research paper associated with this manuscript reported a final sample size of 76. Once corrections are made from comment A1, I would recommend that you specify exactly how many participants’ data was removed owing to technical difficulties and the termination threshold for greater transparency.

B. One of your reuse guidelines is unclear. You mention that “the experimenter was in control of all changes to the VR scenes, especially the gamified visuospatial orientation task could have made use of the augmented motivation, higher engagement, and unconstrained exploration”. This is quite speculative and could use further clarification. I would recommend that you add an operational example of how a researcher might augment motivation or increase engagement. Alternatively, you might want to consider simplifying the guideline.

C1. You have included two DOIs for your data. While the DOI mentioned under heading 3.1 is from 2024, the DOI provided under section 3.9 is a revised version from 2025. Kindly address this by either using the same DOI or explicitly stating that the versions are different.

C2. In the DOI under heading 3.9, there is a preprocessing special cases readme file within the "VR_RFT_RFT" folder which is not mentioned in your manuscript. The manuscript could benefit from a description of said special cases.

Signed, Dhruv Aggarwal
